# Novel *BRCA2* pathogenic variant c.5219 T > G; p.(Leu1740Ter) in a consanguineous Senegalese family with hereditary breast cancer

**DOI:** 10.1186/s12881-019-0814-y

**Published:** 2019-05-06

**Authors:** Jean Pascal Demba Diop, Rokhaya Ndiaye Diallo, Violaine Bourdon-Huguenin, Ahmadou Dem, Doudou Diouf, Mamadou Moustapha Dieng, Seydi Abdoul Ba, Yacouba Dia, Sidy Ka, Babacar Mbengue, Alassane Thiam, Oumar Faye, Papa Amadou Diop, Hagay Sobol, Alioune Dieye

**Affiliations:** 1 Laboratory of Cytology, Cytogenetics and Reproductive Biology, Le Dantec Hospital, Dakar, Senegal; 2 Faculty of Medicine, Pharmacy and Odontology, University Cheikh Anta Diop, Dakar, Senegal; 3 African Center of Excellence for Mother and Child Health (CEA-SAMEF), Dakar, Senegal; 4 Laboratory of Molecular Oncogenetics, Paoli-Calmette Institute, Marseille, France; 5 Joliot Curie Institute, Le Dantec Hospital, Dakar, Senegal; 6 Pasteur Institute of Dakar, Dakar, Senegal

**Keywords:** Hereditary breast cancer, Susceptibility, *BRCA2* gene

## Abstract

**Background:**

Pathogenic variants associated with hereditary breast cancer have been reported for *BRCA1* and *BRCA2 (BRCA1/2)* genes in patients from multiple ethnicities, but limited information is available from sub-Saharan African populations. We report a *BRCA2* pathogenic variant in a Senegalese family with hereditary breast cancer.

**Methods:**

An index case from a consanguineous family and nineteen healthy female relatives were recruited after informed consent. Along with this family, 14 other index cases with family history of breast cancer were also recruited. For the control populations we recruited 48 healthy women with no cancer diagnosis and 48 women diagnosed with sporadic breast cancer without family history. Genomic DNA was extracted from peripheral blood. All *BRCA2* exons were amplified by PCR and sequenced. Sequences were compared to the *BRCA2* GenBank reference sequence (NM_000059.3) using Alamut Software.

**Results:**

We identified a novel nonsense pathogenic variant c.5219 T > G; p.(Leu1740Ter) in exon 11 of *BRCA2* in the index case. The pathogenic variant was also identified in three sisters and one daughter, but was absent in the controls and unrelated cases.

**Conclusions:**

This is the first report of a novel *BRCA2* pathogenic variant in a Senegalese family with hereditary breast cancer. This result confirms the diversity of hereditary breast cancer pathogenic variants across populations and extends our knowledge of genetic susceptibility to breast cancer in Africa.

**Electronic supplementary material:**

The online version of this article (10.1186/s12881-019-0814-y) contains supplementary material, which is available to authorized users.

## Background

Breast cancer is the leading female cancer in the world in terms of incidence and mortality. In 2012 it was diagnosed in 1.7 million cases with 500,000 deaths [1]. Its standardized incidence is estimated at 36.1 per 100,000 women in West Africa [2]. In Senegal, as in most Sub-Saharan African (SSA) countries, breast cancer is the second most common cancer in women after cervical cancer, and may now be the most common cancer based on preliminary reports [2]. It is currently estimated that 5–10% of breast cancers are due to an inherited predisposition [3–5]. Approximately 20–25% of this risk is associated to pathogenic variants of two high penetrance susceptibility genes, *BRCA1* and *BRCA2*, located on chromosomes 17q21 and 13q12, respectively [6, 7]. Both genes are involved in DNA repair and other important biological functions [8]. Risk increases with the number of affected women within the family, early age at diagnosis and the degree of relationship with other affected women [9–11]. The cumulative risk of breast cancer by age 80 years was estimated to 72% for *BRCA1* carriers and 69% for *BRCA2* carriers. For ovarian cancer the cumulative risk by age 80 years was estimated to 44% for *BRCA1* carriers and 17% for *BRCA2* carriers [10–13]. The human *BRCA2* gene contains 27 exons, among which exon 11 is the largest. The coding sequence (Refseq transcript mRNA: NM_000059.3) size is 11,386 bp and it encodes a protein of 3418 amino acids (Refseq protein NP_000050) [14]. Genetic variation analysis of *BRCA2* has identified a large number of different pathogenic germline variants in breast cancer patients and more than a thousand different disease–causing germline pathogenic variants were listed in the Breast Cancer Information Core Database (BIC; http://research.nhgri.nih.gov/bic/) and in the ClinVar database (https://www.ncbi.nlm.nih.gov/clinvar/). Most *BRCA2* pathogenic variants have been reported in individuals of European and Asian origin while limited information is available on SSA populations [15–19]. Herein we report a novel pathogenic variant in *BRCA2* in a consanguineous family with a family history of breast cancer.

## Methods

### Study population

**Fig. 1:**
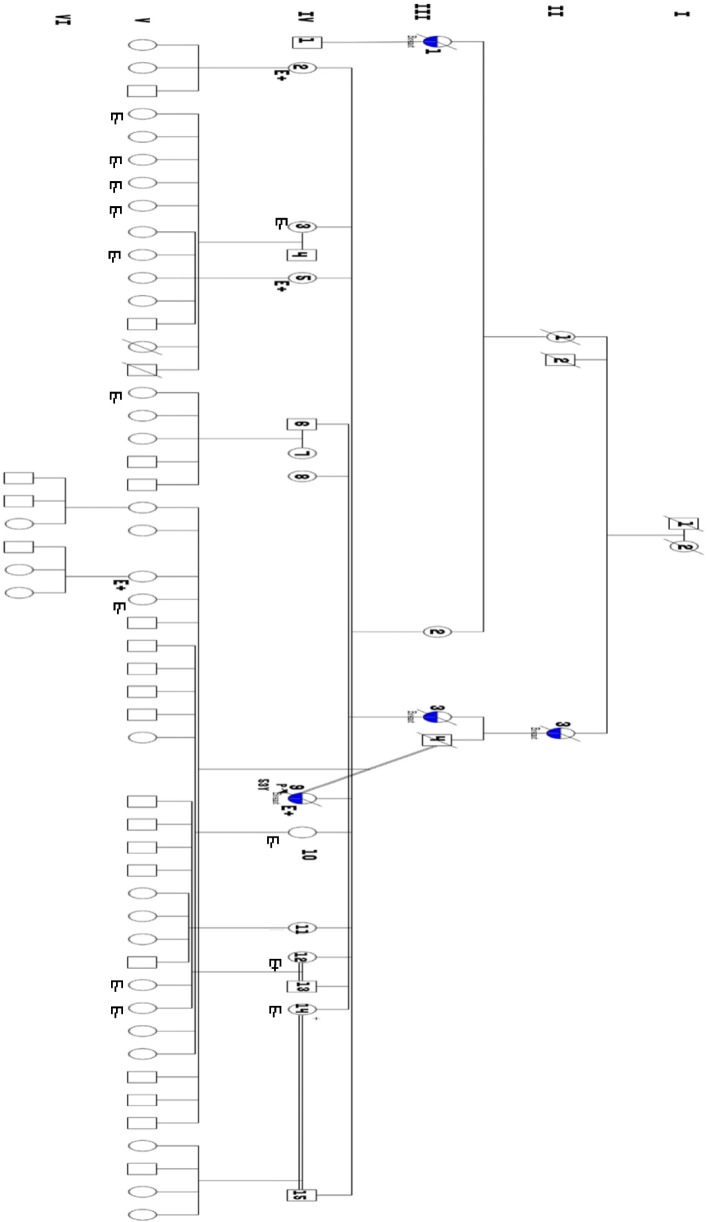
Pedigree and pathogenic variant status of recruited family members

A female index case from a family with a consanguineous mating (Fig. 1) and 19 healthy relative women were recruited after informed consent. The index case was a 53-years-old woman of Wolof ethnicity who died at age 54 from moderately differentiated triple negative ductal adenocarcinoma of the right breast, clinical stage T4dN1M0, SBRII grade. Therapeutic management at the Joliot Curie Institute of Hospital Le Dantec in Dakar, Senegal, consisted of 7 courses of chemotherapy followed by surgical removal of the right breast and radiotherapy. She died as a result of a brain metastasis. Her mother sister, mother’s cousin and maternal grandmother’s sister, also died from breast cancer. The index case was married to her mother’s cousin (Fig. 1**)**. Along with this family, 14 other unrelated index cases with family history of breast cancer, were also recruited. For the control populations we recruited 48 healthy women with no cancer diagnosis (mean age 40.7 years) who came for routine checkup at the Laboratory of Biology of Le Dantec Hospital, and 48 women diagnosed with sporadic breast cancer without family history (mean age at diagnosis 45.7 years) from the Joliot Curie Institute of Le Dantec Hospital. This study was approved by the ethics committee of Cheikh Anta DIOP University under Protocol 014/2014 / CER / UCAD.

### BRCA1 and BRCA2 screening

For each participant a 5 ml blood sample was collected in EDTA tubes. Genomic DNA was extracted from whole blood with a QIAamp® DNA blood Mini kit (Qiagen, Hildel, Germany). In the proband from the consanguineous family as well as in the remaining 14 index cases with familial breast cancer, *BRCA1* and *BRCA2* exons were amplified by PCR with specific primers located in the introns, flanking the intron/exon boundaries. Twenty-eight fragments covering the 22 coding exons of *BRCA1* and 32 fragments covering the 26 coding exons of *BRCA2* gene were amplified [20]. The large exons 10 and 11 of *BRCA2* were amplified as 2 and 9 fragments respectively while exon 11 of *BRCA1* was amplified as 7 fragments (see Additional file 1: Tables S1 and S2). PCRs were carried out with initial denaturation at 95 °C for 10 min followed by 40 cycles of 95 °C for 30s, 55 °C for 30s, and 72 °C for 30s with a GeneAmp® PCR System 9700 (Applied Biosystems) as described previously [20]. The PCR products were purified with a MinElute 96UF kit and sequenced using a Big Dye terminator V3.1 sequencing kit on a 3730 Genetic Analyzer (Applied Biosystems, Foster City, CA, USA). Both forward and reverse strands were sequenced. The sequences were compared to the *BRCA2* GenBank reference sequence (NM_000059.3) with Alamut Software. For the control populations (healthy and sporadic breast cancer groups) and healthy relatives of the proband, only the targeted fragment containing the pathogenic *BRCA2* variant identified in the proband, was sequenced. DNAs from three healthy relatives were of poor quality and were not sequenced.

## Results

**Fig. 2:**
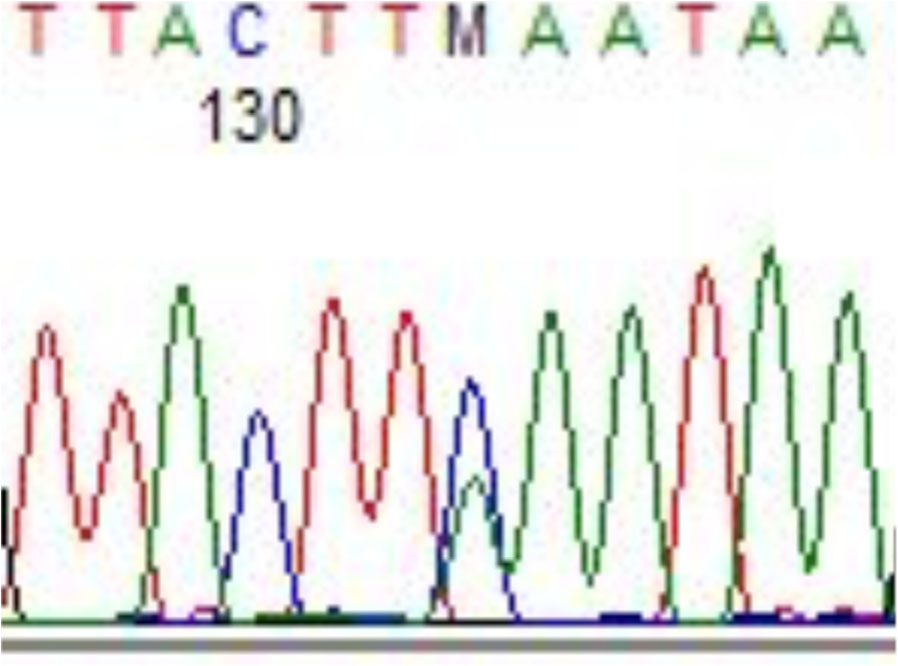
Chromatogram sequence of reverse strand of *BRCA2* exon 11 region surrounding the identified pathogenic variant

No pathogenic variants were identified in *BRCA1* in the proband. A *BRCA2* pathogenic variant, namely c.5219 T > G; p.(Leu1740Ter) (according to the HGVS nomenclature), was identified in the proband. This variant is located in exon 11 of the gene and is predicted to introduce a premature stop codon at position 1740 of the BRCA2 protein. The chromatogram is shown in (Fig. 2).

This pathogenic variant was also detected in three sisters and one daughter of the index case (Fig. 1). The variant was likely transmitted to the index case by her mother (**III-2**) even though she was not diagnosed with breast cancer, but has a sister who died of breast cancer. The index case’s mother in turn would likely have inherited this pathogenic variant from the grandmother (**II-1**) who’s sister died of breast cancer (**II-3**). Four healthy relatives of the index case had the pathogenic variant and would be at risk of developing breast cancer.

This pathogenic variant was not detected in any participant from the two control groups (healthy and sporadic breast cancer groups). This variant has not been described previously in BIC and ClinVar databases, nor in the literature. Other single nucleotide variants were identified in the index case in different *BRCA2* exons **(**Table 1). These variants have been described in the literature and are classified as benign by the expert panel in the ClinVar database and therefore are not pathogenic.

**Table 1:** *BRCA2* variants identified in the index case and classified as benign in ClinVar (RefSeq NM_000059.3)

Variant (HGVS nomenclature)	Effect protein level
c.-26G > A	p.?
c.425 + 67A > C	p.?
c.865A > C	p.(Asn289His)
c.1365A > G	p.(Ser455=)
c.1910-51G > T	p.?
c.2229 T > C	p.(His743=)
c.2971A > G	p.(Asn991Asp)
c.3396A > G	p.(Lys1132=)
c.4563A > G	p.(Leu1521=)
c.6513G > C	p.(Val2171=)
c.7806-14 T > C	p.?
c.8460A > C	p.(Val2820=)
c.9843A > G	p.(Pro3281=)
c.10234A > G	p.(Ile3412Val

For the other 14 index cases recruited for *BRCA1/BRCA2* genetic testing, we identified a recurrent pathogenic variant of the *BRCA1* gene in 6 families out of the 14. No pathogenic variant was detected for the remaining 8 families (data not shown).

## Discussion

Breast cancer is the most commonly diagnosed type of cancer in women in the world [2]. The epidemiology in SSA countries is characterized by younger age at diagnosis, triple negative histopathology, advanced clinical stage and poor prognosis [21–23]. The phenotype of the breast cancer diagnosed in the studied index case matched with this epidemiology. Inherited breast cancer risk is associated to pathogenic variants of two high penetrance susceptibility genes, *BRCA1* and *BRCA2,* yet pathogenic variants in other genes including *PALB2, TP53* and *PTEN* have also been linked with high risk of breast cancer [24, 25]. Pathogenic variants of these genes have been associated with susceptibility to hereditary breast cancer in populations of European and Asian origin [16–18, 26–29] while in Africa, most of the available data come from studies conducted among North African, Nigerian, Sudan and South African populations [16, 30–40].

Few pathogenic variants of *BRCA2* gene have been reported in SSA populations. The *BRCA2* pathogenic variants identified in African populations and reported in the literature are summarized in Table 2. Most causal pathogenic variants have been identified in exon 11 [16, 31, 33, 35–37], and are predominantly deletions or duplications. Only a few nucleotide substitutions leading to premature stop codons have been reported. The novel pathogenic variant we identified in this study is a substitution leading to premature stop codon and is located in exon 11 at position 5219.

**Table 2:** *BRCA2* associated pathogenic variants reported in African populations (RefSeq NM_000059.3)

HGVS nomenclature	Effect protein level	Exon	Country	Reference
c.582G > A	p.(Trp194Ter)	7	South Africa	[16]
c.771_775delTCAAA	p.(Asn257Lysfs	9	Egypt	[38]
c.1528G > T	p.(Glu510Ter)	10	Algeria	[35]
c.1310_1313delAAGA	p.(Lys437Ilefs)	10	Nigeria/Tunisia	[41, 42]
c.1362delA	p.(Lys454Asnfs)	10	Nigeria	[37]
c.5722_5723delCT	p.(Leu1908Argfs)	11	Algeria	[43]
c.6446_6450delTTAAA	p.(Ile2149Serfs)	11	Algeria	[35]
c.3381delT	p.(Phe1127Leufs)	11	Morocco	[39]
c.5073dupA	p.(Trp1692Metfs)	11	Morocco	[44]
c.6428C > A	p.(Ser2143Ter)	11	Morocco	[45]
c.2402_2412delACAATTATGAA	p.(Asn801Ilefs)	11	Nigeria	[37]
c.2808_2811del	p.(Ala938Profs)	11	Nigeria	[37]
c.5130_5133delTGTA	p.(Tyr1710Terfs)	11	Nigeria	[37]
c.5141_5144delATTT	p.(Tyr1714Cysfs)	11	Nigeria	[37]
c.5353_5354delAC	p.(Thr1785Terfs)	11	Nigeria	[37]
c.3195_3198delTAAT	p.(Asn1066Leufs)	11	Soudan	[36]
c.6406_6407delTT	p.(Leu2136Lysfs)	11	Soudan	[36]
c.4798_4800delAAT	p.(Asn1600del)	11	South Africa	[16]
c.5213_5216delCTTA	p.(Thr1738Ilefs)	11	South Africa	[16]
c.5771_5774delTTCA	p.(Ile1924Argfs)	11	South Africa	[16]
c.5946delT	p.(Ser1982Argfs)	11	South Africa	[32]
c.6447_6448dupTA	p.(Lys2150Ilefs)	11	South Africa	[32]
c.6761_6762delTT	p.(Phe2254Tyrfs)	11	South Africa	[33]
c.5681dupA	p.(Tyr1894Terfs)	11	Tunisia	[40]
c.7110delA	p.(Lys2370Asnfs)	13	Morocco	[39]
c.7234_7235insG	p.(Thr2412Serfs)	13	Morocco	[39]
c.7254_7255delAG	p.(Arg2418Serfs)	14	Nigeria	[37]
c.6174delT	p.(Phe2058Leufs)	15	South Africa	[34]
c.7654dupA	p.(Ile2552Asnfs)	16	Algeria/Tunisia	[35, 46]
c.7934delG	p.(Arg2645Asnfs)	17	South Africa	[33]
c.8817_8820delGAAA	p.(Lys2939Asnfs)	22	Nigeria	[37]
c.9097dupA	p.(Thr3033Asnfs)	23	South Africa	[16]
c.9196C > T	p.(Gln3066Ter)	24	Nigeria	[37]

Although other pathogenic variants surrounding this position of the *BRCA2* gene have been reported in the ClinVar database, any pathogenic variant involving the particular codon has been reported in SSA populations (Table 3). These pathogenic variants lead to stop codons or frameshift at amino acid 1739, 1740 or 1741 of BRCA2 protein.

**Table 3:** Reported pathogenic variants in ClinVar at nucleotide 5219 or surrounding this position in *BRCA2* gene (RefSeq NM_000059.3)

Variant (HGVS nomenclature)	Effect protein level
c.5217_5224delTTTAAGTA	p.(Tyr1739Terfs)
c.5217_5223delTTTAAGT	p.(Tyr1739Terfs)
c.5217_5221delTTTAA	p.(Tyr1739Terfs)
c.5217_5220delTTTA	p.(Tyr1739Terfs)
c.5217 T > A	p.(Tyr1739Ter)
c.5218_5234del17	p.(Leu1740Valfs)
c.5218_5224delTTAAGTA	p.(Leu1740Thrfs)
c.5219_5220dup	p.(Ser1741Terfs)
c.5219_5220insTA	p.(Leu1740Phefs)
c.5219dupT	p.(Leu1740Phefs)
c.5219delT	p.(Leu1740Terfs)

Several studies have shown that the pathogenic variant spectrum identified in black populations is different from Caucasian populations [19, 29, 47, 48]. The c.2808_2811delACAA pathogenic variant in *BRCA2* was frequently reported in European populations [49], and was reported only once in a young black girl with breast cancer in Ibadan, Nigeria [27]. The pathogenic variant we identified in *BRCA2* was detected in one family out of 15 recruited for *BRCA*1/2 genetic testing, while we identified a recurrent pathogenic variant of the *BRCA1* gene in 6 families out of the 15 (data not shown). These observations confirm the diversity of pathogenic variants between populations but also within the same population [19, 37, 50].

It has also been reported that Fanconi Anemia (FA) is caused by biallelic *FANCD1/BRCA2* pathogenic variants [51]**.** In this family it was unclear whether there was family member with Fanconi anemia like symptoms or early death, or not.

## Conclusions

We report a novel pathogenic variant c.5219 T > G p.(Leu1740Ter) in *BRCA2* in a consanguineous Senegalese family with a family history of breast cancer. This result highlights the diversity of hereditary breast cancer pathogenic variants and extends the knowledge of genetic susceptibility to breast cancer in Africa. The benefits of clinical genetic testing of *BRCA1/2* in prevention and personalised treatment is unquestionable and it should be adapted to each population’s intrinsic genetic characteristics.

## Additional file

Additional file 1: Ndiaye R BRCA2 Supplementary Material. **Table S1** Primers used for *BRCA1* coding exons PCR amplification. **Table S2** Primers used for *BRCA2* coding exons PCR amplification. (DOCX 26 kb)

## Abbreviations

BIC: Breast Cancer Information Core Database
*BRCA1/2*: Breast cancer gene 1 / 2
DNA: Desoxyribonucleic Acid
EDTA: Ethylene diamine tetra acetic acid
G: Guanine
HGVS: Human Genome Variation Society
NCBI: National Center for Biotechnology Information
PCR: Polymerase Chain Reaction
RefSeq: Reference Sequence
SBR: Scarff-Bloom-Richardson
SNP: Single nucleotide polymorphism
SSA: Sub Saharan Africa
T: Thymine
Ter: Termination stop codon
