# Supplementary material for: Novel BRCA2 pathogenic variant c.5219 T > G; p.(Leu1740Ter) in a consanguineous Senegalese family with hereditary breast cancer
Source: BMC Med Genet. 2019 May 6;20:73. doi: 10.1186/s12881-019-0814-y (PMC6501405; doi:10.1186/s12881-019-0814-y)
Supplement: Supplementary file 1 — Ndiaye R BRCA2 Supplementary Material. Table S1 Primers used for BRCA1 coding exons PCR amplification. Table S2 Primers used for BRCA2 coding exons PCR amplification. (DOCX 26 kb) [file 12881_2019_814_MOESM1_ESM.docx]

**Table 1**: Primers used for BRCA1 coding exons PCR amplification

|  | Primers | |  |
| --- | --- | --- | --- |
| Exon | Primer F | Primer R | Fragment Length (pb) |
| 2 | TGTAAAACGACGGCCAGTGGACGTTGTCATTAGTTCTTTGG | CAGGAAACAGCTATGACCTCTTTTCTTCCCTAGTATGTAAGGTCA | 350 |
| 3 | TGTAAAACGACGGCCAGTTCTCAGTTCCTGACACAGCAG | CAGGAAACAGCTATGACCAAGTTAGGTGTTTCCTGGGTTATG | 315 |
| 5 | TGTAAAACGACGGCCAGTTCATGGCTATTTGCCTTTTG | CAGGAAACAGCTATGACCTCTACTTTTTCCTACTGTGG | 232 |
| 6 | TGTAAAACGACGGCCAGTGGTTTTCTACTGTTGCTGCATCT | CAGGAAACAGCTATGACCCACCACGTCATAGAAAGTAATTGT | 312 |
| 7 | TGTAAAACGACGGCCAGTCACAACAAAGAGCATACATAGGG | CAGGAAACAGCTATGACCCCCAGCTACTAAGGGGGCTA | 370 |
| 8 | TGTAAAACGACGGCCAGTTGTTAGCTGACTGATGATGGT | CAGGAAACAGCTATGACCCACTTCCCAAAGCTGCCTAC | 315 |
| 9 | TGTAAAACGACGGCCAGTCCTGCCACAGTAGATGCTCA | CAGGAAACAGCTATGACCTGCACATACATCCCTGAACC | 312 |
| 10 | TGTAAAACGACGGCCAGTTGGTCAGCTTTCTGTAATCG | CAGGAAACAGCTATGACCGTATCTACCCACTCTCTTTTCAG | 259 |
| 11T1 | TGTAAAACGACGGCCAGTATATAGCCAGTTGGTTGATTT | CAGGAAACAGCTATGACCCATCACTTCTGGAAAACCACTC | 610 |
| 11T2 | TGTAAAACGACGGCCAGTCCTAGAGATACTGAAGATGTTC | CAGGAAACAGCTATGACCGAATAGAATCACCTTTTGTTT | 670 |
| 11T3 | TGTAAAACGACGGCCAGTCAAACGGAGCAGAATGGTC | CAGGAAACAGCTATGACCGACACTTTAACTGTTTCTAG | 621 |
| 11T4 | TGTAAAACGACGGCCAGTAAGTGTTCAAATACCAGTGAAC | CAGGAAACAGCTATGACCATTAGACTCATTCTTTCCTTG | 634 |
| 11T5 | TGTAAAACGACGGCCAGTCACTCTGGGTCCTTAAAGAAAC | CAGGAAACAGCTATGACCCCTAATCTAAGCATAGCATTC | 605 |
| 11T6 | TGTAAAACGACGGCCAGTGAAATAGGTTCCAGTGATG | CAGGAAACAGCTATGACCCAATGATAATAAATTCTCCTC | 620 |
| 11T7 | TGTAAAACGACGGCCAGTTCTCAGTCTACTAGGCATAGC | CAGGAAACAGCTATGACCCTTGGAAATTTGTAAAATGTGC | 494 |
| 12 | TGTAAAACGACGGCCAGTGTCCTGCCAATGAGAAGAAA | CAGGAAACAGCTATGACCTGTCAGCAAACCTAAGAATGT | 280 |
| 13 | TGTAAAACGACGGCCAGTAATGGAAAGCTTCTCAAAGTA | CAGGAAACAGCTATGACCATGTTGGAGCTAGGTCCTTAC | 336 |
| 14 | TGTAAAACGACGGCCAGTTGTGTATCATAGATTGATGCTTTTGA | CAGGAAACAGCTATGACCAAACAAAAGAAGTATCCTAGAGCAA | 394 |
| 15 | TGTAAAACGACGGCCAGTCATGAGCTGTTTCATTTATGCTTT | CAGGAAACAGCTATGACCGAGCTATTTTTCTAAAGTGGGCTTA | 451 |
| 16 | TGTAAAACGACGGCCAGTAATTCTTAACAGAGACCAGAAC | CAGGAAACAGCTATGACCAAAACTCTTTCCAGAATGTTGT | 459 |
| 17 | TGTAAAACGACGGCCAGTAATAGTTCCAGGACACGTG | CAGGAAACAGCTATGACCACCATGCTGGCCAGGATG | 386 |
| 18 | TGTAAAACGACGGCCAGTAGGCTCTTTAGCTTCTTAGGA | CAGGAAACAGCTATGACCAAGACCTTTTGGTAACTCAGA | 290 |
| 19 | TGTAAAACGACGGCCAGTCTGTCATTCTTCCTGTGCTC | CAGGAAACAGCTATGACCCATTGTTAAGGAAAGTGGTGC | 262 |
| 20 | TGTAAAACGACGGCCAGTTGACGTGTCTGCTCCACTTC | CAGGAAACAGCTATGACCCCTGTGTGAAAGTATCTAGCACTG | 310 |
| 21 | TGTAAAACGACGGCCAGTCAGGTGGTGAACAGAAGAAAAA | CAGGAAACAGCTATGACCGAACATTTCAGCAATCTGAGG | 308 |
| 22 | TGTAAAACGACGGCCAGTTCCCATTGAGAGGTCTTGCT | CAGGAAACAGCTATGACCGAACATTTCAGCAATCTGAGG | 308 |
| 23 | TGTAAAACGACGGCCAGTCAGAGCAAGACCCTGTCTC | CAGGAAACAGCTATGACCACTGTGCTACTCAAGCACCA | 312 |
| 24 | TGTAAAACGACGGCCAGTCCTGGAGTCGATTGATTAGAGC | CAGGAAACAGCTATGACCGAAGGACTGAAGAGTGAGAGGAG | 404 |

**Table 2:** Primers used for BRCA2 coding exons PCR amplification

|  | Primers | |  |
| --- | --- | --- | --- |
| Exon | Primer F | Primer R | Fragment Length (pb) |
| 2 | TGTAAAACGACGGCCAGTTCCAGCGCTTCTGAGTTTTA | CAGGAAACAGCTATGACCGGAACAGTTTATGGTTCTAAGCAA | 316 |
| 3 | TGTAAAACGACGGCCAGTGGGTCACAAATTTGTCTGTC | CAGGAAACAGCTATGACCGATTTTAACACAGGTTTGCCT | 390 |
| 4 | TGTAAAACGACGGCCAGTACTCCCTATACATTCTCATTC | CAGGAAACAGCTATGACCTCTTCTACCAGGCTCTTAG | 354 |
| 5_7 | TGTAAAACGACGGCCAGTGTAGTATTCCAACAATTTAT | CAGGAAACAGCTATGACCCAACCTCATCTGCTCTTTCTTG | 790 |
| 8 | TGTAAAACGACGGCCAGTTCAATTCATTTTGTTTCAAATGTGT | CAGGAAACAGCTATGACCCCAGGTTTAGAGACTTTCTCAAAGG | 324 |
| 9 | TGTAAAACGACGGCCAGTGAAATCACCAAAAGTGAAACCA | CAGGAAACAGCTATGACCGGGTGACAGAGCAAGACTCC | 270 |
| 10T1 | TGTAAAACGACGGCCAGTTGTTTCTATGAGAAAGGTTGTGAGA | CAGGAAACAGCTATGACCTTGCCTGCTTTACTGCAAGA | 710 |
| 10T2 | TGTAAAACGACGGCCAGTGAATTCTTTGCCACGTATTTCT | CAGGAAACAGCTATGACCAAAAACACAGAAGGAATCGTCA | 662 |
| 11T1 | TGTAAAACGACGGCCAGTCACTGTGCCCAAACACTACC | CAGGAAACAGCTATGACCAGATTTGTGTTTTGGTTGAATTG | 710 |
| 11T2 | TGTAAAACGACGGCCAGTTCCCATGGAAAAGAATCAAGA | CAGGAAACAGCTATGACCTTCAACACAAGCTAAACTAGTAGGAT | 670 |
| 11T3 | TGTAAAACGACGGCCAGTGCAGGACTCTTAGGTCCAATTT | CAGGAAACAGCTATGACCTGTGCCATGAGCAGAATAAA | 710 |
| 11T4 | TGTAAAACGACGGCCAGTGACAGCAGCAAGCAATTTGA | CAGGAAACAGCTATGACCTGTTCTTTATTTGAAGTATTACCATGA | 709 |
| 11T5 | TGTAAAACGACGGCCAGT TGGCCAGTTTATGAAGGAGG | CAGGAAACAGCTATGACCACTTTGGGGCAGCTGTGAT | 710 |
| 11T6 | TGTAAAACGACGGCCAGTTTTTGATGAAAAAGAGCAAGG | CAGGAAACAGCTATGACCTTCTTCAATACTGGCTCAATACCA | 778 |
| 11T7 | TGTAAAACGACGGCCAGTCAAAAATCATCTCTCCGAAAAA | CAGGAAACAGCTATGACCGAAACTTTCTCCAATCCAGACA | 660 |
| 11T8 | TGTAAAACGACGGCCAGTAGGTTGTTACGAGGCATTGG | CAGGAAACAGCTATGACCCCATTTCTGAGTTTACACAGTGC | 669 |
| 11T9 | TGTAAAACGACGGCCAGTCAGCAAGTGGAAAGCAAGTTT | CAGGAAACAGCTATGACCCACAAAAATGGCTGAAAAGACA | 680 |
| 12 | TGTAAAACGACGGCCAGTCTCTTTCAAACATTAGGTCACTAT | CAGGAAACAGCTATGACCCAGCACTTTGAGAGGCAGGT | 356 |
| 13 | TGTAAAACGACGGCCAGTGTGTATTTACAGTAACATG | CAGGAAACAGCTATGACCGTTAACTTCTTAACGTTAG | 261 |
| 14 | TGTAAAACGACGGCCAGTGAGGGTCTGCAACAAAGG | CAGGAAACAGCTATGACCCATCAGGACATTATTTAAC | 604 |
| 15 | TGTAAAACGACGGCCAGTGCCAGGGGTTGTGCTTTTTAA | CAGGAAACAGCTATGACCTCATTCATCCATTCCTGCACTA | 380 |
| 16 | TGTAAAACGACGGCCAGTGGTAAATTCAGTTTTGGTTTGTTA | CAGGAAACAGCTATGACCGAAAGAGGGATGAGGGAATACATA | 353 |
| 17 | TGTAAAACGACGGCCAGTAAAACTTAATGATCTTGAACAATG | CAGGAAACAGCTATGACCGCAACATTTTGACATGGAAGTC | 383 |
| 18 | TGTAAAACGACGGCCAGTTTTTATTCTCAGTTATTCAGTGAC | CAGGAAACAGCTATGACCTTGAGCATCCTTAGTAAGCATTT | 550 |
| 19 | TGTAAAACGACGGCCAGTGGCAGTTCTAGAAGAATGAAAACTC | CAGGAAACAGCTATGACCGCTGCAGTGAACCAAGATCA | 392 |
| 20 | TGTAAAACGACGGCCAGTTGCCTGGCCTGATACA | CAGGAAACAGCTATGACCCCCTTGTTGCTATTCTTTGT | 409 |
| 21 | TGTAAAACGACGGCCAGTGGGTGTTTTATGCTTGGTTCT | CAGGAAACAGCTATGACCTCCTGTGATGGCCAGAGAGT | 303 |
| 22 | TGTAAAACGACGGCCAGTAACCACACCCTTAAGATGAGC | CAGGAAACAGCTATGACCGGGCATTAGTAGTGGATTTTGC | 404 |
| 23_24 | TGTAAAACGACGGCCAGTTGCCCACAAAGAGATAATATAAAAGA | CAGGAAACAGCTATGACCTGCCAACTGGTAGCTCCAAC | 603 |
| 25 | TGTAAAACGACGGCCAGTCATATTAGAGTTTCCTTTCTTGCAT | CAGGAAACAGCTATGACCTTCCTTGATACTGGACTGTCAAAA | 410 |
| 26 | TGTAAAACGACGGCCAGTGGTCCAAACTTTTCATTTCTGC | CAGGAAACAGCTATGACCGGAAAGTGTGCACCCAGAGT | 463 |
| 27 | TGTAAAACGACGGCCAGTGATTTAGTTTTTTATGTTACTAC | CAGGAAACAGCTATGACCGTACTAATGTGTGGTTTG | 660 |
